# The Sperm Olfactory Receptor OLFR601 is Dispensable for Mouse Fertilization

**DOI:** 10.3389/fcell.2022.854115

**Published:** 2022-06-03

**Authors:** González-Brusi L, Hamzé JG, Lamas-Toranzo I, Jiménez-Movilla M, Bermejo-Álvarez P

**Affiliations:** ^1^ Animal Reproduction Department, INIA-CSIC, Madrid, Spain; ^2^ Department of Cell Biology and Histology, Medical School, IMIB, University of Murcia, Murcia, Spain

**Keywords:** fertilization, sperm, TMEM95, olfactory receptor, odorant

## Abstract

Fertilization involves the fusion of two gametes by means of yet unknown membrane binding and fusion events. Over the last years, many sperm proteins have been uncovered to play essential roles in sperm-egg fusion in mammals, but their precise role in fertilization remains unknown, being unclear how these proteins interact with each other or with other yet unknown sperm proteins. The aim of this study has been to identify possible sperm proteins interacting with TMEM95, a protein essential for fertilization located in the sperm membrane. A list of 41 sperm proteins that were pulled down with TMEM95 and identified by mass spectrometry did not include other sperm proteins known to play a role in fertilization, suggesting an independent role of TMEM95 in fertilization. Between these lists, OLFR601 is allocated to the acrosomal region and may mediate affinity for an odorant involved in fertilization. However, *Olfr601* disruption did not impair the sperm fertilization ability, suggesting that its function may be redundant with that of other sperm proteins.

## 1 Introduction

Sexual reproduction requires the binding and fusion of two gametes during fertilization. Despite being arguably the most relevant cell fusion event in a mammal’s life, until very recently, only three proteins were proven to be essential for this process: two sperm membrane proteins [IZUMO1 (Inoue et al., 2005) and SPACA6 (Lorenzetti et al., 2014)] and JUNO, a protein located on the oolemma (i.e., oocyte membrane) (Bianchi et al., 2014). The ablation of CD9, another oolemma-located protein, significantly impairs fertilization and it has been suggested to play a structural role (Kaji et al., 2000; Le Naour et al., 2000; Miyado et al., 2000). The list of known proteins required for this process has grown considerably on the sperm side with the addition of other sperm proteins including TMEM95, FIMP, SOF1, and DCST1/2. TMEM95 ablation was reported to cause severe infertility (Pausch et al., 2014) and *in vitro* fertilization defects in bulls (Fernandez-Fuertes et al., 2017), and complete fertilization failure in mice (Lamas-Toranzo et al., 2020; Noda et al., 2020). In mice, FIMP ablation significantly reduces fertilization rates (Fujihara et al., 2020) and, similar to TMEM95, SOF1, DCST1, or DCST2 ablation prevents fertilization (Noda et al., 2020; Inoue et al., 2021). The specific role of these sperm proteins on gamete fusion is evidenced by the remarkably similar reproductive phenotype of their corresponding KOs: sperms lacking any of these proteins are morphologically and kinetically normal, being able to reach the perivitelline space while failing to ultimately fuse their membrane with that of the oocyte.

Despite the exciting momentum, it remains unknown how these newly discovered sperm proteins interact to mediate gamete fusion and whether other proteins are required. Any cell fusion event, including fertilization, requires the first step of membrane binding before membrane fusion. IZUMO1-JUNO binding remains the only known protein-to-protein interaction between the sperm membrane and the oolemma (Bianchi et al., 2014), and IZUMO1 is still the only sperm protein described to be involved in the binding step, as sperms lacking any of the other six sperm proteins (SPACA6, TMEM95, SOF1, FIMP, DCST1, and DCST2) show binding to the oolemma of zona-devoid oocytes to a similar degree as the WT counterparts (Fujihara et al., 2020; Lamas-Toranzo et al., 2020; Noda et al., 2020; Inoue et al., 2021). Although sperm binding assays may not be fully conclusive in mice because of unspecific sperm binding, the lack of known interactions between any of the other six sperm proteins and JUNO or any other oocyte protein further suggests that they may be involved in post-binding events ultimately leading to gamete fusion. In this perspective, uncovering the interactions between these and other sperm proteins may help solve the fertilization puzzle.

The shared localization of some of the proteins involved in fertilization within the sperm allows protein-to-protein interactions. IZUMO1 and TMEM95 are localized to the acrosomal region in acrosome intact sperms and relocate to the head, including the equatorial segment, after an acrosome reaction (Satouh et al., 2012; Lamas-Toranzo et al., 2020). Similar localization patterns in acrosome-intact and acrosome-reacted sperms were observed for SPACA6 in human sperm, although their localization could not be confirmed in mice (Barbaux et al., 2020). In partial contrast to IZUMO1, TMEM95, and SPACA6, FIMP localizes to the sperm head, including the equatorial segment before an acrosome reaction, being faintly detected in that region in ∼60% of the acrosome-reacted sperm (Fujihara et al., 2020). Finally, the localization of SOF1, DCST1, and DCST2 is unknown, although a membrane localization is unlikely for SOF1 because it lacks a transmembrane domain. Interestingly, IZUMO1 localization is not affected by the ablation of any of the other sperm proteins involved in fertilization (Barbaux et al., 2020; Fujihara et al., 2020; Lamas-Toranzo et al., 2020; Noda et al., 2020; Inoue et al., 2021), suggesting that they play IZUMO1-independent roles in fertilization. However, SPACA6 is absent in sperms lacking DCST1, DCST2, or IZUMO1, evidencing possible interactions or relations between these four proteins (Inoue et al., 2021). The aim of this study has been to explore sperm proteins interacting with TMEM95 and to characterize the fertilization ability of sperms lacking one of these proteins: OLFR601.

## 2 Materials and Methods

### 2.1 Production of Recombinant TMEM95 Protein

Two recombinant TMEM95 proteins were produced, one containing the transmembrane domain (TMD, 147-167 aa, Uniprot P0DJF3) and another lacking that domain (TMEM95∆Tm). The lack of a transmembrane domain facilitates its secretion and recovery, so TMEM95∆Tm was used to perform the pull-down assay. Expression plasmids [pcDNA3.1 (+)] were designed and constructed to encode mouse TMEM95 protein (UniProt P0DJF3) (GeneArt). A histidine tag (HHHHHH) was added to the C-terminus of TMEM95 and TMEM95∆Tm. In addition, a FLAG-tag (DYKDDDDK) was added in position 27-34 aa to both constructs. After verification by Sanger sequencing, Tmem95 and Tmem95∆Tm expression plasmids were amplified using Library Efficiency DH5αTM Competent cells (Thermo Fisher Scientific) and purified with a GenEluted Plasmid Kit. Chinese Hamster Ovary cells (CHO cells, the European Collection of Authenticated Cell Cultures (ECACC)) were grown (37°C, 5% CO_2_, and 95% humidity) for 48–72 h to 80–90% confluence using an F-12 medium (Biowest) supplemented with 10% fetal bovine serum (Biowest) and 100 U/mL penicillin–streptomycin (Gibco). Transient transfections were performed with Lipotransfectina (Solmeglas), adding 4 µL of the reagent to a final volume of 200 µL Opti-MEM reduced-serum medium (Gibco) containing plasmids and incubated for 15 min at room temperature (RT). The complex was diluted by adding 2 ml Opti-MEM and overlaid on growing cells (37°C, 5% CO_2_, and 95% humidity). The medium containing the secreted proteins was collected after 48 h, centrifuged at 4,000 g for 5 min at 4°C to remove cell debris, and concentrated in Vivaspin® Turbo 4 of 10,000 Da (Sartorius). A final volume of 200–300 µL of concentrated proteins was obtained in 20 mM sodium phosphate buffer, pH 7.4 with a protease inhibitor (EDTA-free EASYpack, Roche). A cell-growing medium containing concentrated proteins was separated by SDS-PAGE and transferred to PVDF membranes which were probed with the primary antibodies anti-Flag (Sigma F7425) and anti-TMEM95 (MyBioSource MBS7004333), both at 1:1,000 v/v in TBST 1X, 1% BSA, before visualization by chemiluminescence (Pierce ECL-Plus, Thermo Fisher Scientific).

#### 2.1.1 Conjugation of Recombinant TMEM95ΔTm to Magnetic Beads

Magnetic Sepharose^®^ beads (His Mag Sepharose Excel™; GE Healthcare) were homogenously resuspended and 20 µL of bead slurry was pipetted into a micro-centrifuge tube containing 500 µL of 20 mM sodium phosphate buffer, pH 7.4. The beads were washed with 500 µL of washing buffer (20 mM sodium phosphate, 0.5 M NaCl, 10 mM imidazole, pH 7.4) and finally in 500 µL of binding buffer (20 mM sodium phosphate, 0.5 M NaCl, 0.1% Tween-20, pH 7.4). Concentrated recombinant TMEM95∆Tm was incubated with washed magnetic beads (1:1 v/v) overnight at 4°C with orbital agitation. After incubation, the beads coated with protein (B_TMEM95∆Tm_) were washed twice with 20 mM sodium phosphate buffer (pH 7.4) to remove non-conjugated proteins. Then, they were resuspended in 20 mM sodium phosphate buffer pH 7.4 and solubilized under reducing conditions in 4X SDS sample buffer (Millipore, United States). After 10 min at 100°C, the supernatant was separated by SDS-PAGE, transferred to PVDF membranes, and probed with the anti-TMEM95 (MyBioSource, United States) as mentioned previously.

#### 2.1.2 Pull-Down Assay

WT sperms were collected from cauda epididymis in PBS and centrifuged at 3,000 g for 7 min. Pellets were snap-frozen in liquid nitrogen and kept at −80°C until analysis. The sperm pellets were suspended in 400 µL of solubilization buffer (50 mM Tris-HCl pH 7.5, 1 mM EDTA, 1% Igepal, 0.1 mM PMSF, 10 mM iodoacetamide, 10 mM N-ethylmaleimide, phosphatase inhibitor, and protease inhibitor). The sample was centrifuged at 15,000 g for 30 min and the supernatant was co-incubated with unconjugated Sepharose^®^ beads (B) or Sepharose® beads conjugated with TMEM95 recombinant protein as described previously (B_TMEM95∆Tm_) at 4°C with orbital agitation overnight. After incubation, unconjugated or conjugated Sepharose^®^ beads (B and B_TMEM95∆Tm_) were recovered and non-specific proteins bound to B (B + sperm) or TMEM95-interacting proteins bound to B_TMEM95∆Tm_ (B_TMEM95∆Tm+sperm_) were eluted adding 50 µL of elution buffer (20 mM sodium phosphate, 0.5 M NaCl, 500 mM imidazole, pH 7.4) and incubated in agitation at 4°C for 1 h. This procedure was repeated three times obtaining a final volume of 150 µL. A control for bead conjugation was also included by eluting B_TMEM95∆Tm_ beads, not incubated in the presence of sperm lysates (B_TMEM95∆Tm_).

The eluted fractions (B + sperm, B_TMEM95∆Tm+sperm_, and B_TMEM95∆Tm_) were processed for proteomic analysis at a molecular biology service (SACE, University of Murcia, Spain). The protein identity was determined by mass spectrometry, carried out by using an HPLC/MS system composed of an Agilent 1290 Infinity II Series HPLC (Agilent Technologies) equipped with an automated multi-sampler module and a high speed binary pump, and connected to an Agilent 6550 Q-TOF Mass Spectrometer (Agilent Technologies, Santa Clara, CA, United States) using an Agilent Jet Stream Dual electrospray (AJS-Dual ESI) interface. Experimental parameters for HPLC and Q-TOF were set in MassHunter Workstation Data Acquisition software (Agilent Technologies, Rev. B.08.00). Data processing and analysis were performed using a Spectrum Mill MS Proteomics Workbench (Rev B.06.00.201, Agilent Technologies).

Proteins identified by mass spectrometry were listed for B, B_TMEM95∆Tm+sperm_, and B_TMEM95∆Tm_. A Venn diagram was used to detect sperm proteins that specifically interacted with recombinant TMEM95ΔTm (http://bioinformatics.psb.ugent.be/webtools/Venn). This list was curated to detect potential proteins involved in fertilization by applying two selection criteria: 1) absence of a fertile KO reported, based on the information of http://www.informatics.jax.org and 2) membrane localization, based on gene ontology information available in UniProt (cellular component).

#### 2.1.3 Generation of *Olfr601* KO

All experimental procedures were approved by the INIA ACUC committee and Madrid Region Authorities (PROEX 040/17) in agreement with European legislation. sgRNA was designed against a sequence (ACA​GAG​CAT​GCG​TGG​CAA​TG) at the beginning of the coding region of *Olfr601* (NM_146314.2) using bioinformatics tools to minimize the chances of an off-target genome edition (https://crispr.mit.edu). sgRNA was synthesized and purified using a Guide-it sgRNA *In Vitro* Transcription Kit® (Takara). Capped polyadenylated Cas9 mRNA was generated by *in vitro* transcription (mMESSAGE mMACHINE T7 ULTRA kit®, Life Technologies) using as a template the plasmid pMJ920 (Addgene 42234) linearized with BbsI (NEB). mRNA was purified using a MEGAClear kit (Life Technologies).

C57CBAF1 female mice 7–8 weeks old were super-ovulated by intraperitoneal injections of 5 IU of pregnant mare serum gonadotropin (PMSG, Folligon®, MSD Animal Health) and an equivalent dose of human chorionic gonadotropin (hCG, Sigma) at a 48-h interval. The super-ovulated female mice were mated with C57CBAF1 stud males and zygotes were recovered from oviducts. Microinjections were performed with a micromanipulation system (Eppendorf TransferMan 4r and Femptojet 4i) equipped with a Leica DMi8 inverted microscope. A mixture of 150 ng/µL of Cas9 mRNA and 50 ng/µL of sgRNA was delivered into the cytoplasm of the zygotes (3–5 pL) using a filament needle (Bermejo-Álvarez et al., 2015).

After microinjection, the embryos were cultured in EmbryoMax® KSOM Mouse Embryo Media (Millipore) at 37°C under 5% CO_2_ for 4 days until they reached the blastocyst stage and were transferred to a pseudo-pregnant Swiss recipient 2.5 days post-coitum (dpc). Genotyping was performed using primers spanning the target sequence (F: 5′-CAC​GAG​CCC​ATG​TTC​CTC​TT-3′, R: 5′-CAC​AGA​TGG​CCA​CAT​AGC​GA-3′, 217 bp product in WT) under the following conditions: 95°C for 2 min; 35 × (94°C for 20 s, 60°C for 30 s, and 72°C for 30 s); 72°C for 5 min; hold at 8°C. The PCR products from F0 mice were purified using a FavorPrep™ PCR Purification Kit (Favorgen), cloned into pMD20 T-vectors (Takara) using Blunt TA ligase (NEB), and transformed into *Escherichia coli* DH5-α competent cells. A total of 10 positive plasmid clones from each transformation were purified (Favorgen) and Sanger-sequenced (Stabvida) to uncover the alleles generated after the CRISPR-mediated edition harbored by each individual. Following genotyping, a founder female carrying two KO alleles composed of 1 and 10 bp deletions was crossed with C57BL/6 WT males to obtain heterozygous mutants. Heterozygous F1 individuals carrying the 10 bp deletion were intercrossed to produce WT, Hz, or KO individuals used for the experiments (Figure 2C). Subsequent generations were genotyped by a quantitative PCR high-resolution melting (qPCR-HRM) curve analysis that allowed the detection of WT, Hz, and KO individuals. qPCRs were performed on a Mic qPCR cycler (BioMolecular Systems) with primers flanking the target sequence (F 5′-TGA​CCT​GGT​CCT​CTC​CAC​AT-3′, R 5′-AAG​GCA​TGC​GTC​GAA​GGT​AA-3′, 88 bp product in WT, 78 bp on the KO allele). Reaction conditions were as follows: 40 × (94°C for 15 s, 56°C for 30 s, and 72°C for 20 s). Melting curves were visualized using Mic qPCR hardware (BioMolecular Systems) and contrasted with those obtained from known WT, Hz, and KO samples confirmed by Sanger sequencing.

#### 2.1.4 RNA Analysis

Transcriptional analysis was performed as previously described. Briefly, total RNA was collected from testis, seminal vesicle, prostate, and epididymis samples (three samples/tissue) obtained from WT males using Trizol (Invitrogen). After DNAse treatment (Promega), RNA was retro-transcribed (qScript Quantabio) to cDNA. *Olfr601* and *Gapdh* transcripts were detected on cDNA by PCR using the amplification cycle described in genotyping. Primers to detect *Olfr601* were the same as those used for HRM-based genotyping (217 bp product). Primers used for *Gapdh* were F 5′-ACC​CAG​AAG​ACT​GTG​GAT​GG-3′ and R 5′-ATG​CCT​GCT​TCA​CCA​CCT​TC-3′ (247 bp). Primers used to detect the expression of other OLFRs are listed in Supplementary Table S1. DNAse-treated non-retro-transcribed RNA obtained from the testis served as a negative control for DNAse treatment and PCR.

#### 2.1.5 Sperm Immunocytochemistry

Sperms from WT or KO individuals were recovered from the cauda epididymis in the HTF medium and incubated for 1 h at 37°C in a 5% CO2 water-saturated atmosphere. Following incubation, the sperms were centrifuged (3,000 g for 7 min), washed with PBS, fixed in 4% paraformaldehyde in PBS for 5 min, and washed twice in PBS. The samples were then permeabilized with 0.1% Triton X-100 in PBS and blocked with 5% FCS in PBS for 45 min at 4°C. Next, the samples were incubated with primary antibodies overnight at 4°C. The primary antibodies used were anti-OLFR601 (rabbit polyclonal custom-made, CliniSciences, 1:100), anti-TMEM95 antibody (rabbit polyclonal, MyBioSource, 1:100), and anti-IZUMO1 (mouse monoclonal KS139-34, a gift from Dr. Ikawa). After washing, the samples were incubated with secondary antibodies for 2 h at RT. The secondary antibodies used were donkey anti-rat IgG Alexa Fluor 647 and donkey anti-rabbit IgG Alexa Fluor 488 (1:500, Invitrogen). Finally, the samples were incubated for 5 min with 1 µg/ml lectin-PNA Alexa Fluor 568 (Invitrogen) and 1 µg/ml Hoechst 33342 (Sigma). They were then mounted and subsequently observed under an epifluorescence inverted microscope (Zeiss Axio Observer) equipped with structured illumination (Zeiss Apotome).

#### 2.1.6 OLFR601 Immunoblotting

WT and KO sperms were collected from the cauda epididymis in PBS supplemented with 0.1% PVP and centrifuged at 3,000 g for 7 min. Pellets were snap-frozen in liquid nitrogen and kept at −80°C until analysis. The frozen pellets were re-suspended in Laemmli buffer (4X) and boiled for 10 min (lysis protocol #1) in a lysis buffer composed of 50 mM Tris HCl (pH8), 10 mM DTT, and 2% SDS boiled for 10 min with vigorous agitation (lysis protocol #2), in M-PERTM Mammalian Protein Extraction Reagent supplemented with HaltTM Protease Inhibitor Cocktail (100X) (Thermo Fisher Scientific) for 1 h at 4°C with vigorous agitation (lysis protocol #3) or RIPA buffer supplemented with HaltTM Protease Inhibitor Cocktail (100X) for 1 h at 4°C with vigorous agitation (lysis protocol #4). After incubation, the samples were centrifuged at 8,200 g for 10 min to discard cell debris. The supernatant containing sperm proteins was separated by SDS-PAGE and transferred to PVDF membranes which were probed with two custom polyclonal rabbit antibodies (anti-OLFR601-1 and anti-OLFR601-2, 1:1000 v/v in TBST 1X 1% BSA) before visualization by chemiluminescence (Pierce ECL-Plus, Thermo Fisher Scientific).

### 2.2 Fertility Tests

#### 2.2.1 *In vivo* Fertilization Analysis

Initial fertility tests were performed on three males per genotype (WT, Hz, and KO) that were co-caged with WT 7–8 weeks old C57CBAF1 females (1:1 ratio). Mating was assessed by the presence of a copulatory plug and each male was allowed to mate twice. Twenty-one days after mating, the pups were counted to annotate the litter size. Statistical differences were analyzed by ANOVA–taking all six data/group and by performing a Wilcoxon rank-sum exact test–taking the average litter size of each male when data were not normal (Shapiro–Wilk test) or homoscedastic (Levene test) (SigmaStat package) and the level of significance was set at *p* < 0.05.

*In vivo* fertilization analysis was performed using WT and KO males and 7–8 week-old C57CBAF1 females. Female mice were super-ovulated as described previously and mated with WT or KO males (three individuals/group, two females/male tested). Embryos were recovered from the oviduct on 1.5 dpc and the cleavage rate was assessed upon recovery. Statistical differences were analyzed by ANOVA–taking all six data/group and by Wilcoxon rank-sum exact test–taking the average litter size of each male when data were not normal (Shapiro–Wilk test) or homoscedastic (Levene test) (SigmaStat package) and the level of significance was set at *p* < 0.05.

*In vitro* fertilization analysis was performed using WT and KO males and 7–8 week-old C57CBAF1 females**.** Sperms from WT or KO individuals (three individuals/group) were recovered from the cauda epididymis in an HTF medium and placed in the bottom of a previously equilibrated 300 µL drop of HTF covered with mineral oil for 2 h before IVF. After pre-incubation time, the upper 150 µL of the drop was collected and the spermatozoa concentration was analyzed. Cumulus-oocyte complexes (COCs) were recovered from the oviduct of super-ovulated female mice 14 h after hCG injection and placed in a 4-well dish with 400 µL of human tubal fluid (HTF) medium in groups of ∼40 COCs per well. Previously prepared spermatozoa were immediately added to the well containing COCs at a final concentration of 10^6^ spermatozoa/ml. After 4 h of co-incubation, presumptive zygotes were sequentially washed in an M2 and KSOM medium and cultured as described previously. A fusion assay was performed on the COCs collected as described (14 h after hCG injection) and the cumulus cells were removed by incubation in 300 µg/ml hyaluronidase solution in an M2 medium. Zona pellucida was removed by brief incubation in an acidic Tyrode’s medium and zona-free mouse oocytes were pre-incubated in HTF with Hoechst 33342 1 µg/ml for 10 min and washed before sperm addition. After 30 min of gamete co-incubation (as mentioned previously), the oocytes were fixed in a 0.25% glutaraldehyde solution in PVS and observed under fluorescence microscopy. Statistical differences were analyzed by a Wilcoxon rank-sum exact test (SigmaStat package) and the level of significance was set at *p* < 0.05.

## 3 Results

**FIGURE 1:**
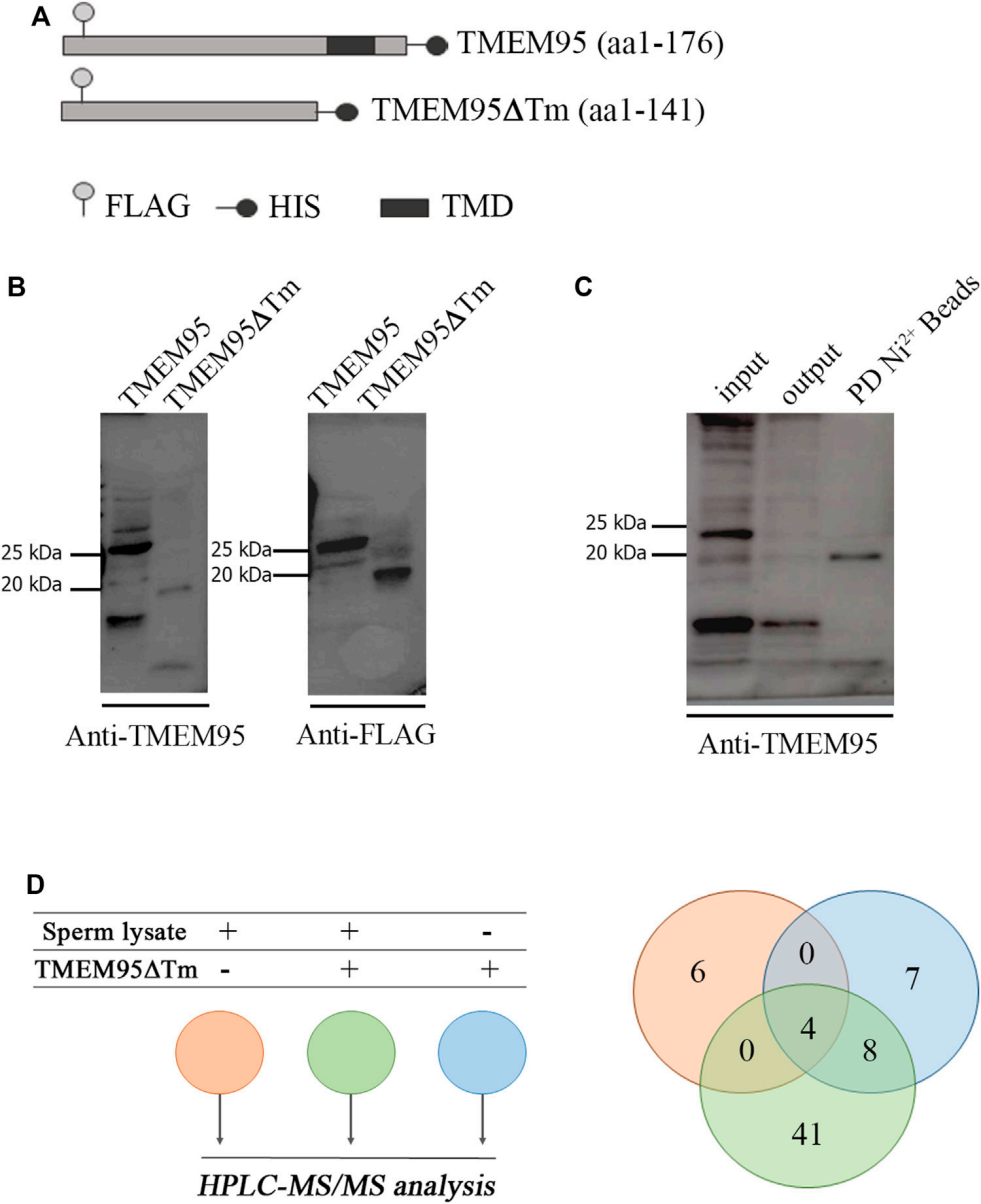
Expression of recombinant TMEM95 and pull-down. **(A)** Schematic representation of recombinant TMEM95 proteins. FLAG sequence (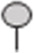), histidine tail (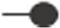), and transmembrane domain (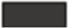) indicated. **(B)** Proteins were expressed in CHO cells, separated by SDS-PAGE and analyzed by Western blot. TMEM95 and TMEM95∆Tm were probed with anti-TMEM95 and anti-FLAG antibodies. **(C)** SDS-PAGE and Western blot of BTMEM95∆Tm. Input: medium with secreted TMEM95∆Tm before conjugation. Output: media after conjugation. IP:Ni+2 Beads: eluted fraction containing TMEM95∆Tm. **(D)** Venn diagram from the listed sperm proteins identified by HPLC-MS/MS analysis. Proteins from the sperm lysate that bind non-specifically to Sepharose® beads (pink), proteins eluted from BTMEM95∆Tm (blue), and specifically bound to TMEM95∆Tm (i.e., sperm proteins interacting with TMEM95, green, Supplementary Table S1).

A pull-down assay performed on sperm lysates with beads conjugated with recombinant TMEM95 was used to uncover sperm proteins potentially interacting with TMEM95. Expression plasmids encoding TMEM95 and TMEM95∆Tm proteins (Figure 1A) were expressed in Chinese hamster ovary (CHO) cells and secreted proteins were successfully isolated. Each protein had the expected molecular mass. TMEM95-recombinant proteins showed a molecular weight of 25 kDa and TMEM95∆Tm (lacking the transmembrane domain) was 20 kDa on immunoblots probed with anti-Flag and anti-TMEM95 antibodies (Figure 1B). After incubation of beads with a medium containing secretions from transfected CHO cells, recombinant TMEM95∆Tm protein was successfully conjugated to beads (Figure 1C).

The pull-down assay revealed a list of 41 sperm proteins identified by mass spectrometry (see Materials and Methods) seemingly interacting with TMEM95 (Figure 1D, Supplementary Table S2). These proteins cannot be deemed as *bona fide* TMEM95 interactors, as the pull-down assay is not completely reliable to identify interactors and requires further validation. The sperm proteins known to play relevant roles during fertilization (IZUMO1, SPACA6, SOF1, DCST1, and DCST2) were not present on that list. Nine of the proteins on the list were located on the membrane according to gene ontology information available in UniProt (Cellular Component): TRIP10, LIFR, IGHV5-16, GAPDH, OLFR601, CALCR, NID1, LRGUK, and EEF1A1. From this reduced list of nine proteins, two (CALCR and LRGUK) were reported to be localized to the acrosomal vesicle. Previous publications on KO models for these two proteins observed that *Calcr* ablation did not affect male reproductive function (Davey et al., 2008), whereas *Lrguk* ablation causes male infertility, as the protein is a major determinant of the microtubule structure within the male germline (Liu et al., 2015). TRIP10 (also known as CIP4) constitutes another interesting candidate involved in gamete fusion, as it is a Cdc-42 interacting protein involved in actin dynamics and podosome formation (Linder et al., 2000), and podosome-like structures have been involved in myoblast fusion (Sens et al., 2010). Unfortunately, *Trip10* ablation does not cause infertility in mice (Feng et al., 2010; Koduru et al., 2010) so its putative role in fertilization is unclear and could be masked by the redundant function of other genes. In this sense, after curation based on available KO models, only two of the membrane-localized sperm proteins potentially interacting with TMEM95 (IGHV5-16 and OLFR601) were not explored by gene ablation experiments before. IGHV5-16 belongs to a family of immunoglobulin heavy variables located in mouse chromosome 12 accounting for 216 genes plus other pseudo-genes and 239 alleles, which makes its deletion technically challenging. In contrast, OLFR601 is a single copy gene encoding for an olfactory receptor with an unknown function. The gene does not have a direct human ortholog but shares a ∼55% identity with human OR52M1, a similar percentage of identity to other proteins involved in fertilization such as IZUMO1 (Supplementary Table S3). Unfortunately, attempts to prove TMEM95-OLFR601 interaction by co-immunoprecipitation were unsuccessful because of the lack of a suitable antibody against OLFR601 for WB analysis (Supplementary Figure S1).

**FIGURE 2:**
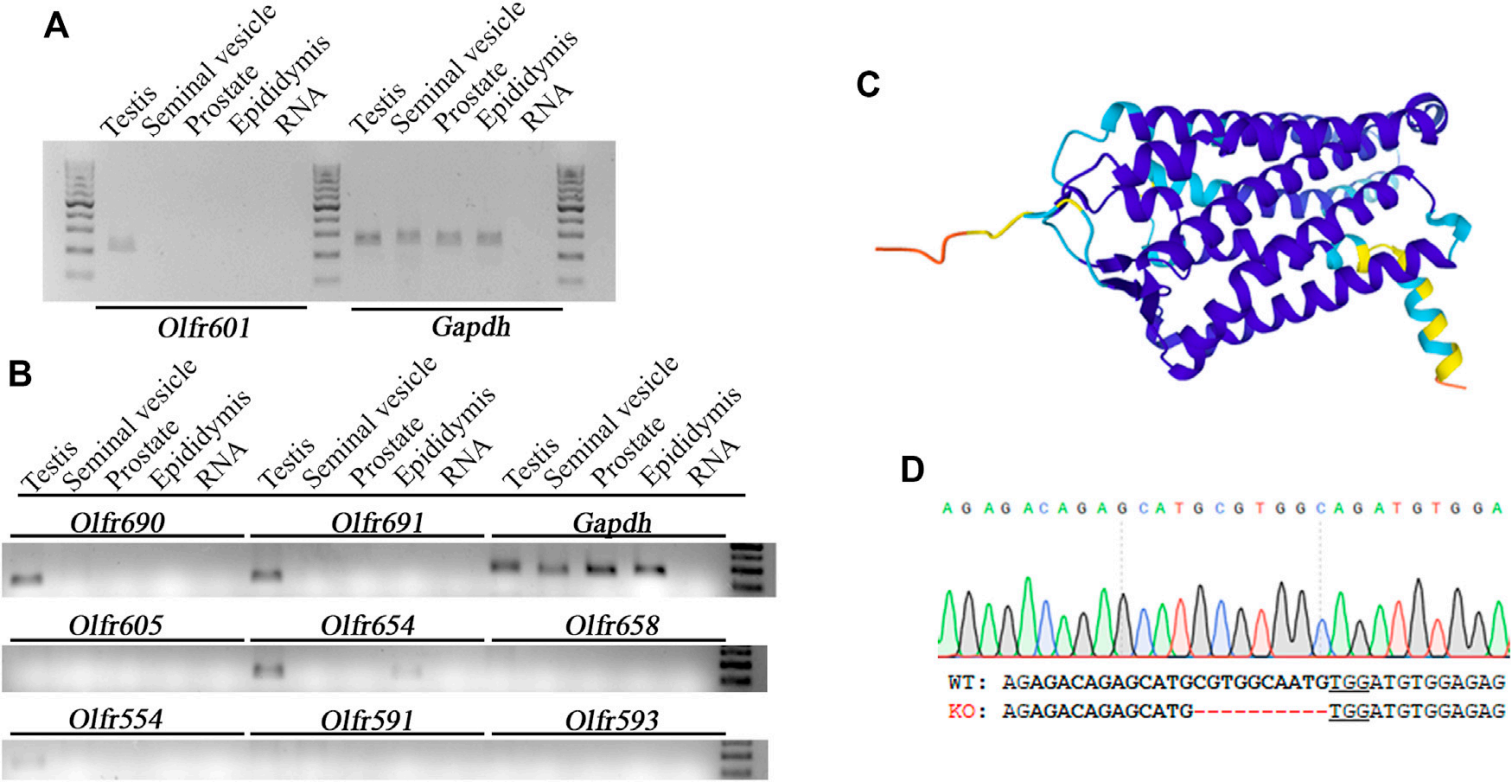
Expression pattern, structure, and ablation details of OLFR601. **(A)** *Olfr601* mRNA is expressed exclusively in the testis (T), being undetectable in the seminal vesicles (s.v.), prostate (Pr), or epididymis (Ep). **(B)** mRNA expression of different olfactory receptors showing ∼50–90 sequence identity with *Olfr601*. **(C)** OLFR601 shows a typical 3D structure of an olfactory receptor, being composed of seven helix and three unconnected beta strands according to the Alphafold model Uniprot (A2RS33). **(D)** Details of the deletion of 10 bp generated by CRISPR, selected as KO alleles to generate the mouse line. Lower letters indicate the DNA sequence for WT and KO alleles. Bold letters indicate the CRISPR target site and the PAM sequence is underlined.

*Olfr601* mRNA was present specifically in the testes, being absent in other male reproductive tissues such as the seminal vesicles, prostate, or epididymis (Figure 2A). The mRNA expression of other eight olfactory receptors displaying ∼50–90% sequence identity with OLFR601 (Supplementary Table S1) was also assessed, showing that three of them (*Olfr690*, *Olfr691,* and *Olfr554*) were also exclusively expressed in the testis, whereas another (*Olfr654*) was expressed in the testis and epididymis (Figure 2B). The predicted tri-dimensional structure of OLFR601, obtained by AlphaFold (Varadi et al., 2021) was composed of seven transmembrane helices and three unconnected beta strands (Figure 2C), a typical structure from olfactory receptors displaying an affinity for diverse types of molecules. As such structures may drive OLFR601 affinity for an odorant, not necessarily a protein, present in the sperm or in the oolemma, we sought to determine the role of this protein in fertilization by loss-of-function experiments.

**FIGURE 3:**
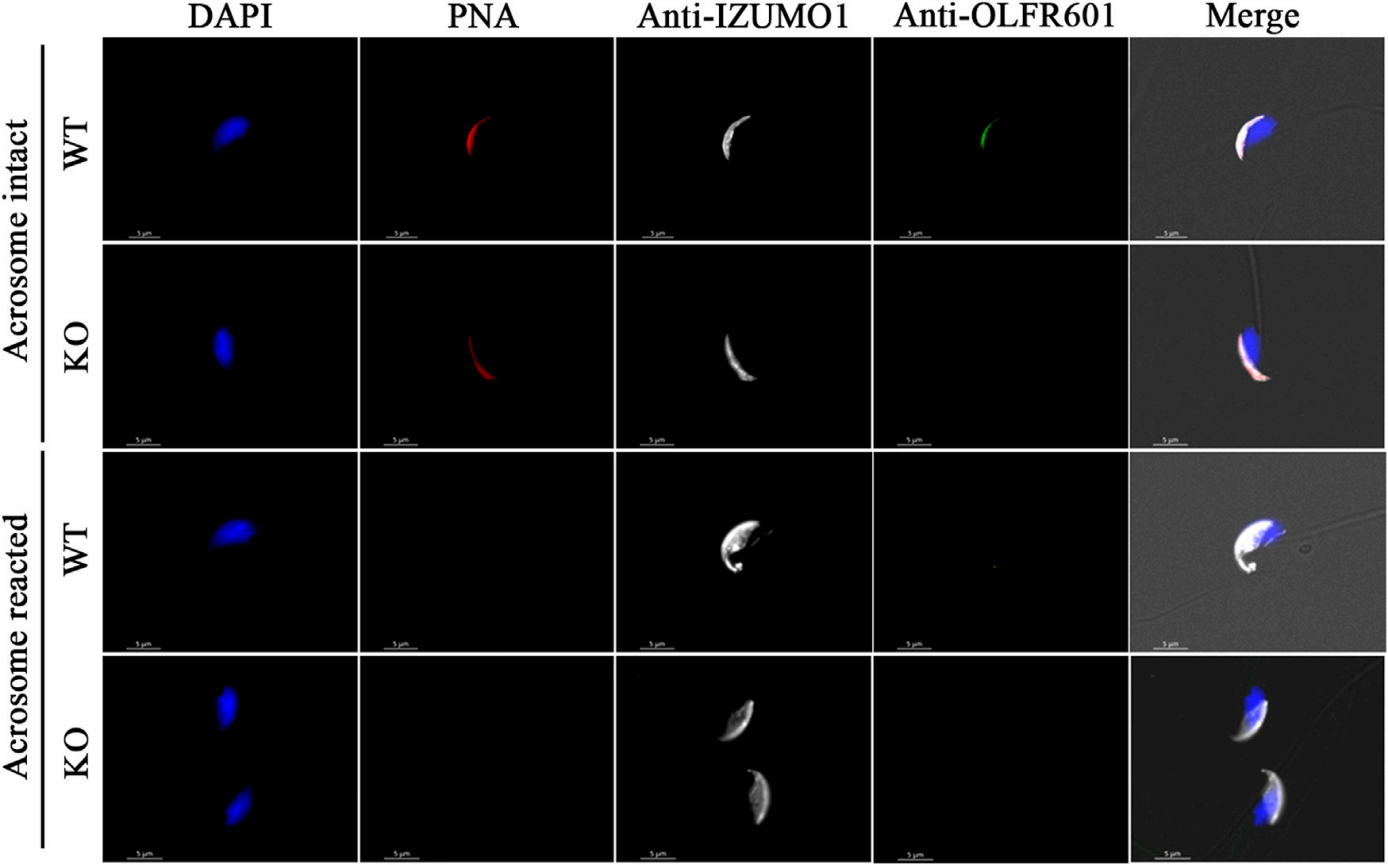
Localization of OLFR601 and IZUMO1 in acrosome-intact or acrosome-reacted WT or *Olf601* KO sperm. Sperm nuclei were stained with DAPI (blue) and acrosomes by PNA staining (red). IZUMO1 (white) localization was not affected by *Olfr601* ablation. OLFR601 (green) localizes to the acrosomal region in acrosome-intact sperm, being undetectable in acrosome-reacted sperm. No OLFR601 protein was detected on KO sperms.

**FIGURE 4:**
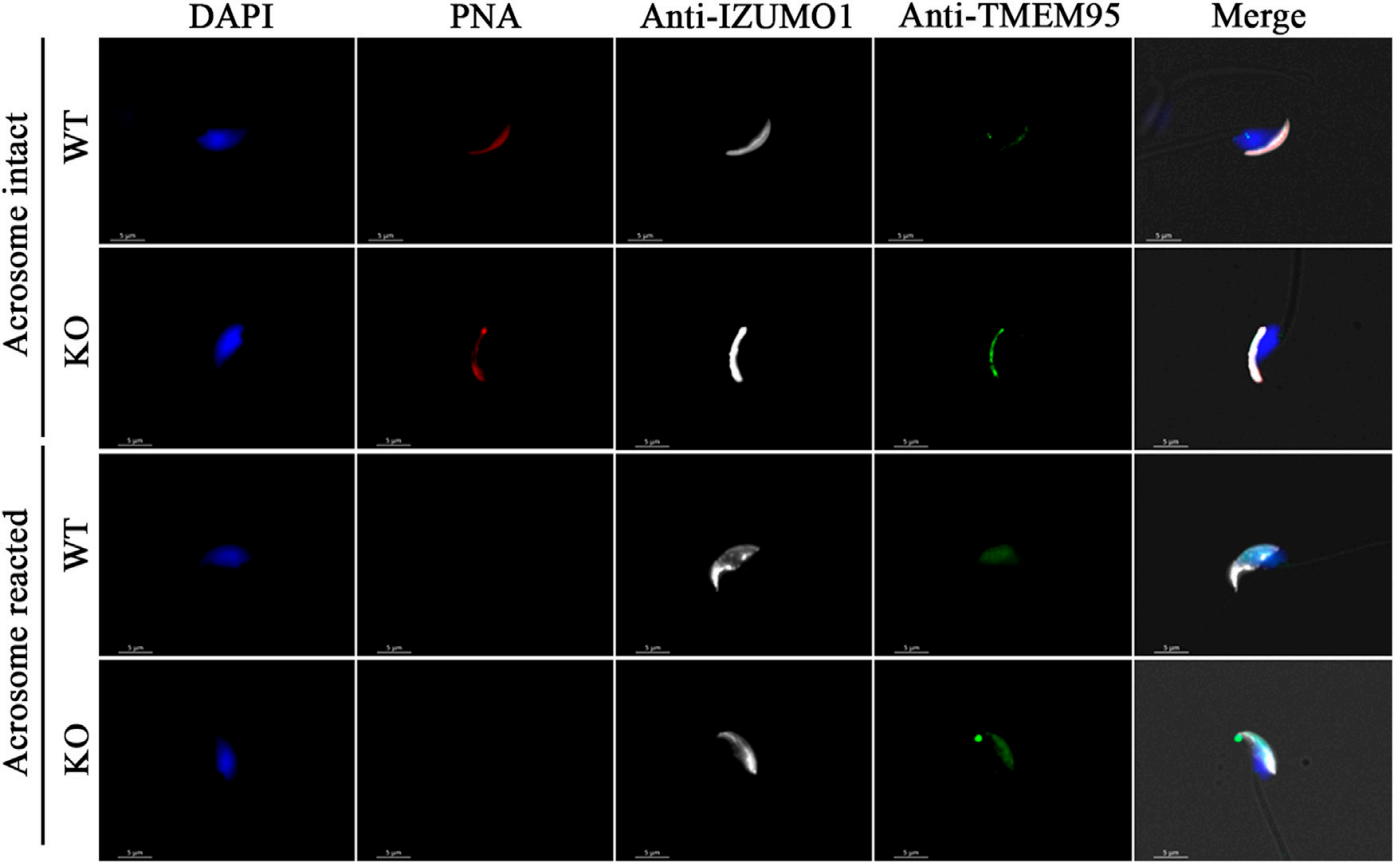
Localization of IZUMO1 and TMEM95 in acrosome-intact or acrosome-reacted WT or *Olfr601* KO sperms. Sperm nuclei were stained with DAPI (blue) and acrosomes by PNA staining (red). The localization of IZUMO1 (white) and TMEM95 (green) was not affected by *Olfr601* ablation.

Breeding of a mouse line harboring a KO allele composed of a 10 bp deletion that results in a 79-amino-acid-truncated peptide (Figure 2D) revealed no deviation from the Mendelian inheritance pattern, evidencing that OLFR601 plays no essential role during development. Protein ablation was confirmed by immunocytochemistry. OLFR601 was present in the acrosomal region of almost all (98/100) acrosome-intact WT sperms, becoming undetectable after the acrosome reaction (Figure 3 and Supplementary Figure S2). As expected, no OLFR601 was detected in acrosome-intact or acrosome-reacted KO sperm. The disruption of OLFR601 did not alter IZUMO1 localization to the acrosomal region in acrosome-intact WT or KO sperm and to the head, including the equatorial segment, in acrosome-reacted WT or KO sperm. Similarly, sperms lacking OLFR601 did not show altered TMEM95 localization patterns, which localizes to the acrosomal region in acrosome-intact sperm and translocates to the head, including the equatorial segment, after the acrosome reaction in WT or KO sperms (Figure 4). The OLFR601 localization pattern was not affected in sperms lacking TMEM95 (Lamas-Toranzo et al., 2020) (Supplementary Figure S3).

**FIGURE 5:**
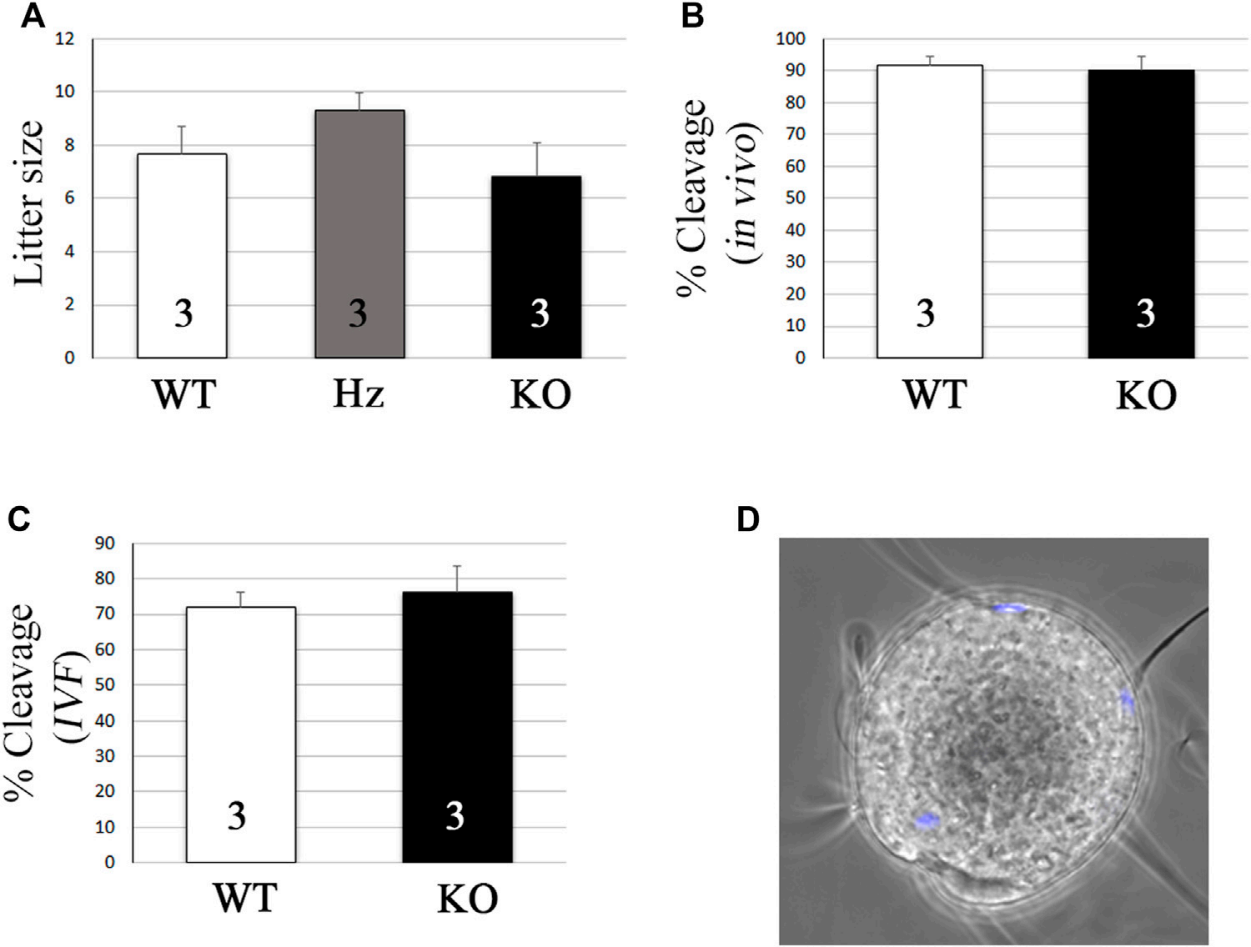
*Olfr601* KO sperms are able to fertilize eggs. **(A)** Litter size after mating of WT or *Olfr601* KO males with WT females. Columns display mean ± s.e.m. Numbers within each column indicate the number of males tested. ANOVA *p* > 0.05. **(B)** *In vivo* fertilization rates after mating of WT or *Olfr601* KO males with super-ovulated WT females. Columns display mean ± s.e.m. Numbers within each column indicate the number of males tested. ANOVA *p* > 0.05. **(C)** *In vitro* fertilization rates following co-incubation of WT or *Olfr601* KO sperms and WT oocytes. Columns display mean ± s.e.m. Numbers within each column indicate the number of males tested. ANOVA *p* > 0.05. **(D)** Sperm-egg fusion assay. Sperm lacking OLFR601 fused with Hoechst pre-loaded zona-free eggs, which transferred the stain to them upon membrane fusion.

KO individuals were overtly normal and KO males showed normal mating behavior and were able to father litters of a comparable size to their Hz or WT siblings (Figure 5A, 7.7 ± 1 vs. 9.3 ± 0.6 vs. 6.8 ± 1.3 pups, mean ± s.e.m. for WT, Hz, and KO, respectively). As expected, KO females were also fertile (data not shown). To further test if fertilization was impaired, *in vivo* and *in vitro* fertilization tests were performed to compare WT and KO sperms. *In vivo* fertilization rates using super-ovulated females were similar after mating with WT or KO males (Figure 5B, 91.6 ± 2.9 vs. 90.2 ± 4.2% cleavage, mean ± s.e.m. for WT and KO, respectively). Similarly, *in vitro* fertilization rates did not vary using WT or KO sperms (Figure 5C, 78 ± 13.3 vs. 71.8 ± 7% cleavage, mean ± s.e.m. for WT and KO, respectively). Finally, as expected based on the unaltered *in vivo* and *in vitro* fertility, OLFR601 KO sperms were able to fuse with zona-free eggs (Figure 5D).

## 4 Discussion

Despite the recently enlarged list of known sperm proteins essential for fertilization, the understanding of the binding and fusion processes behind fertilization has not advanced significantly since the discovery of JUNO (Bianchi et al., 2014), as IZUMO1:JUNO protein interaction remains the only known protein-to-protein interaction proven to be involved in mammalian fertilization. Given that IZUMO1 is involved in gamete binding through its interaction with JUNO (Bianchi et al., 2014) and that none of the other sperm proteins seems to play an essential role in gamete binding (Fujihara et al., 2020; Lamas-Toranzo et al., 2020; Noda et al., 2020; Inoue et al., 2021), the specific roles in fertilization of the other sperm proteins seemingly take place on post-binding events. However, cell fusion assays combining TMEM95, SPACA6, SOF1, IZUMO1, and JUNO found that they are not sufficient to induce cell fusion (Lamas-Toranzo et al., 2020; Noda et al., 2020), suggesting that other unknown proteins or molecules are required for membrane fusion. In this perspective, exploring the relations between these and other sperm proteins could uncover protein complexes directly or indirectly required for gamete fusion. The list of sperm proteins potentially interacting with TMEM95 based on the pull-down assay did not include IZUMO1, SPACA6, FIMP, SOF1, DCST1, or DCST2, that is, the sperm proteins known to be involved in fertilization. While the location of some of these proteins remains unknown, IZUMO1 and SPACA6 are present in similar sperm regions to TMEM95 before and after the acrosome reaction, and also share a similar predicted structure, thereby allowing possible interactions that may have not been detected by the pull-down assay. However, IZUMO1 localization is not affected by *Tmem95* ablation (Lamas-Toranzo et al., 2020), whereas, although *Spaca6* ablation does not alter IZUMO1 localization either (Noda et al., 2020), no SPACA6 protein can be detected after *Izumo1*, *Dcst1,* or *Dcst2* ablation (Inoue et al., 2021). These findings suggest that while SPACA6, IZUMO1, DCST1, and DCST2 roles are seemingly related, TMEM95 may play a role not directly related to the other sperm proteins known to be required for fertilization.

Although the list of sperm proteins pulled down with TMEM95 included diverse cytosolic and nuclear proteins, probably resulting from non-specific binding due to the low salt conditions (see Material and Methods), two of the proteins potentially interacting with TMEM95 -CALCR and LRGUK- were previously known to localize to the acrosomal region, a localization pattern compatible with a role in fertilization. The ablation of CALCR did not affect male fertility (Davey et al., 2008), proving that it does not play an essential role in fertilization. In contrast, LRGUK plays an essential role in the basal body and manchette function during spermatogenesis (Liu et al., 2015). As LRGUK is involved in sperm morphology re-modeling, it may play a role in re-modeling events occurring after gamete binding, however, exploring such a possibility would require dedicated investigation, as the altered morphology of *Lrguk* KO sperms prevents fertilization before membrane binding. Other than these two acrosomal proteins, OLFR601 was the most promising candidate to be involved in fertilization as, although the protein was not detected after the acrosome reaction, its acrosomal localization in acrosome-intact sperm coincides with TMEM95, IZUMO1, SPACA6, and FIMP.

OLFR601 displays a typical structure of an olfactory receptor, often associated with an affinity for specific molecules (odorant), not necessarily proteins. The possible involvement of odorants in fertilization may open a novel interesting perspective to uncover unknown molecules required for the process. Given that OLFR601 is lost after the acrosome reaction, its putative intervention and that of its interacting odorant during gamete fusion can only be indirect, maybe mediating the formation of complexes of proteins required for gamete fusion. However, as OLFR601 disruption does not affect any step of fertilization, it may either or not play any role in the process or play a redundant function with other receptors. In agreement with the latter possibility, the other three olfactory receptors sharing ∼50% sequence identity with OLFR601 were also exclusively expressed in the testis. Olfactory receptors are known to be expressed in organs and tissues outside the olfactory epithelium, including the testes (Parmentier et al., 1992), which are the richest olfactory receptor-expressing tissues excluding the olfactory epithelium (Massberg and Hatt, 2018). Up to 91 olfactory receptors have been reported to be expressed in human and mouce sperm (Flegel et al., 2015) and several have been found to play roles in chemotaxis. In human sperm, the olfactory receptor OR1D2 localizes to the mid-piece and plays a role in sperm chemotaxis (Spehr et al., 2003). Mouse OR267-12 (OLFR16) has also been suggested to play a role in sperm chemotaxis (Fukuda et al., 2004) and OR7A5 and OR4D1 have been suggested to influence sperm motility (Veitinger et al., 2011). In any case, although the absence of an infertility phenotype following *Olfr601* ablation proves that it is not essential for fertilization, it does not exclude that the ablation may have caused some subtle alterations in the sperm that could be detected by a deeper functional analysis.

In conclusion, the ablation of the olfactory receptor OLFR601 does not disrupt fertilization, suggesting that it either does not play a role in fertilization or it plays an indirect role in gamete fusion which may be redundant with that of other proteins.

## Data Availability

The original contributions presented in the study are included in the article/Supplementary Material; further inquiries can be directed to the corresponding authors.

## Supplementary Material

Supplementary Figure S1 Attempts to detect the OLFR601 protein on the SDS-page were unsuccessful after different lysis protocols using two custom-made antibodies. OLFR601-1 is the antibody showing specific staining in ICC (Figure 3 and Supplementary Figure S2).

Supplementary Figure S2 Representative pictures of multiple acrosome-intact (PNA+) WT sperm showing consistent acrosome localization of both IZUMO1 and OLFR601. PNA-sperm loss OLFR601 while maintaining IZUMO1.

Supplementary Figure S3 Representative pictures of acrosome-intact (PNA+) or acrosome-reacted (PNA-) *Tmem95* KO sperm. Sperms lacking TMEM95 show normal localization of OLFR601. OLFR601 localizes to the acrosomal region before the acrosome reaction and it is not detected after acrosome reaction.

Supplementary Table S1 Details for primers used to amplify transcripts from olfactory receptors showing ∼50–90 protein identity with OLFR601.

Supplementary Table S2 Sperm proteins potentially interacting with TMEM95 according to the pull-down assay.
