# Supplementary figures and images for: The Sperm Olfactory Receptor OLFR601 is Dispensable for Mouse Fertilization
Source: Front Cell Dev Biol. 2022 Jun 3;10:854115. doi: 10.3389/fcell.2022.854115 (PMC9204177; doi:10.3389/fcell.2022.854115)

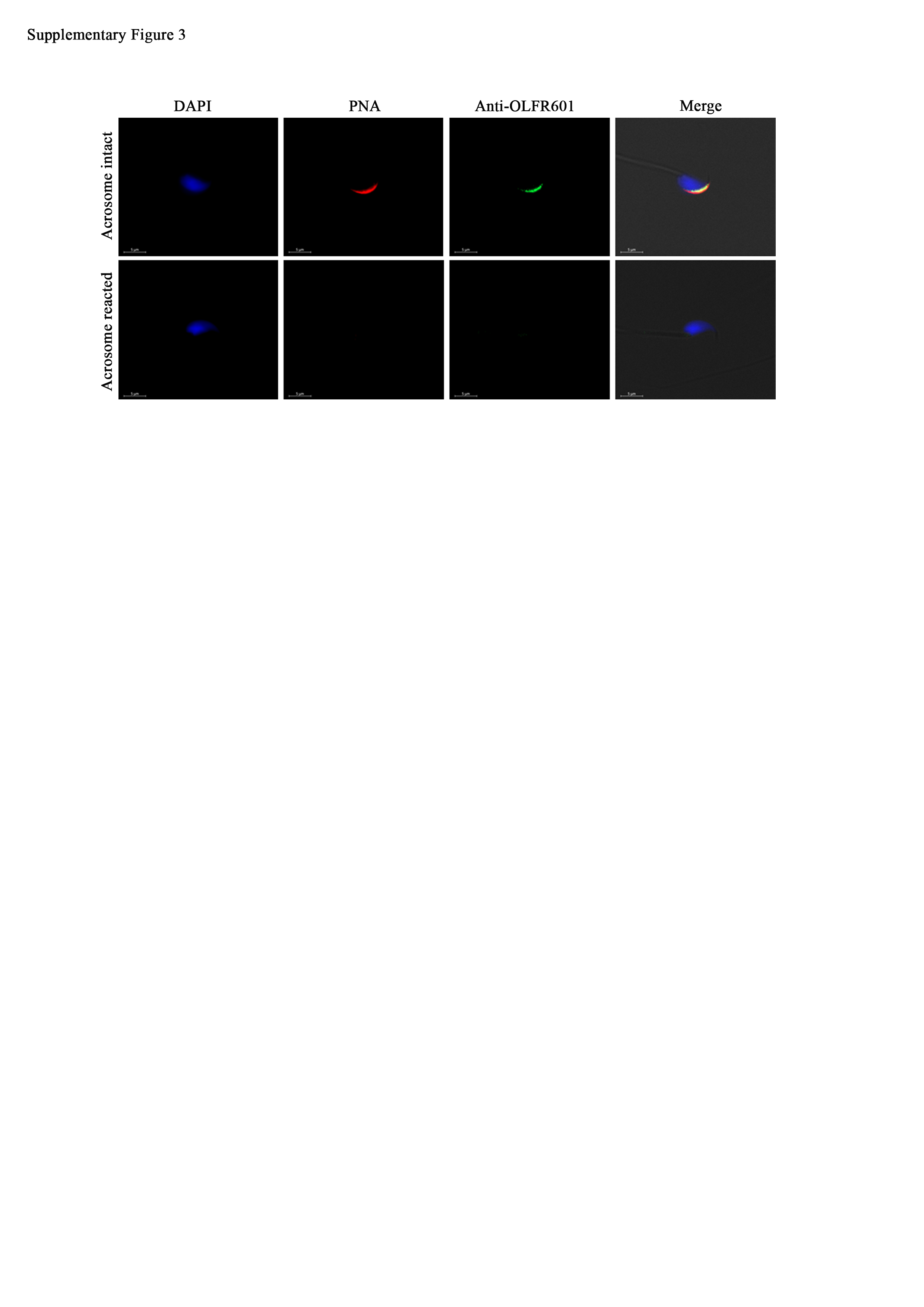

Supplement: Supplementary file 2 [file Image3.TIF]

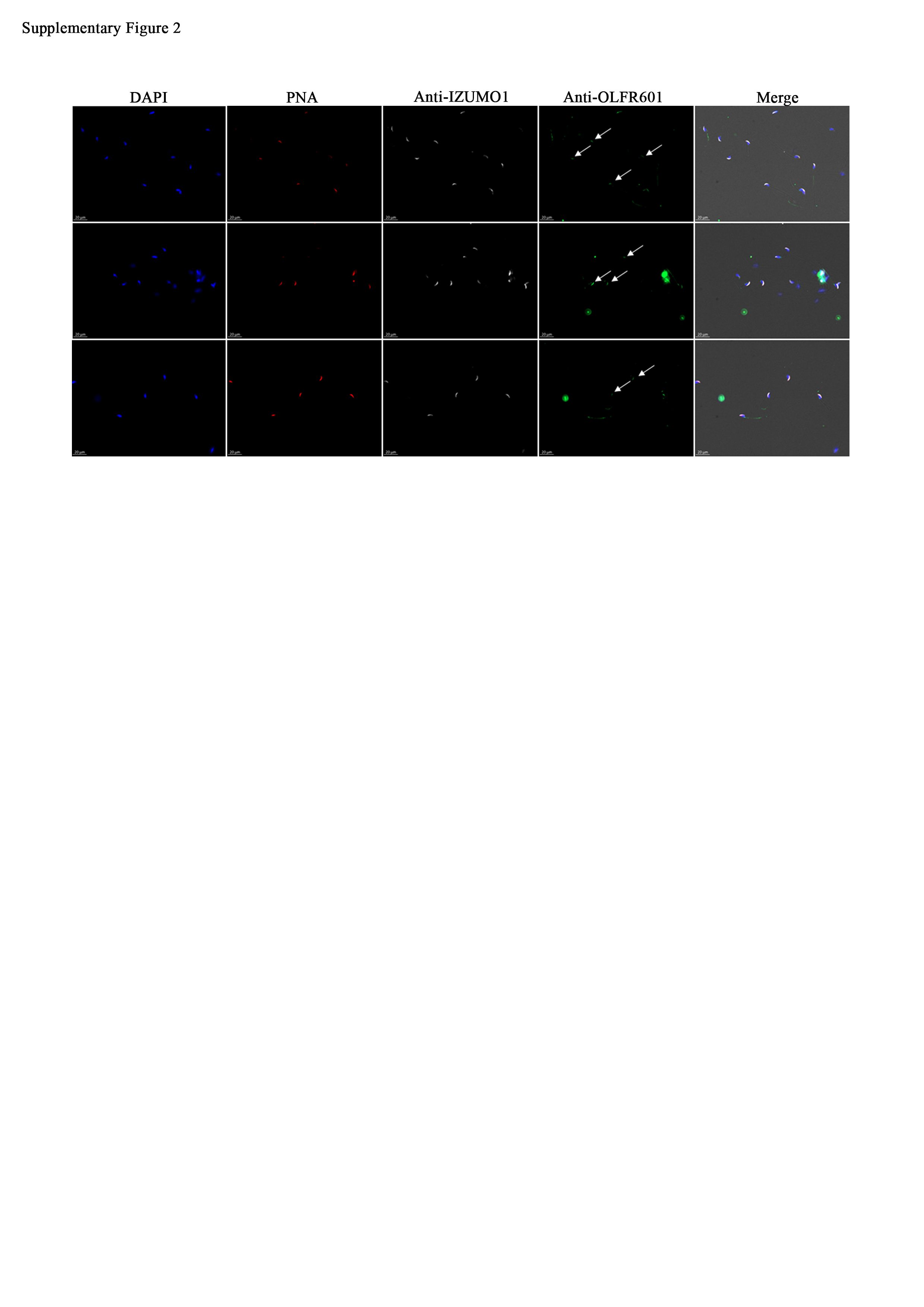

Supplement: Supplementary file 3 [file Image2.TIF]

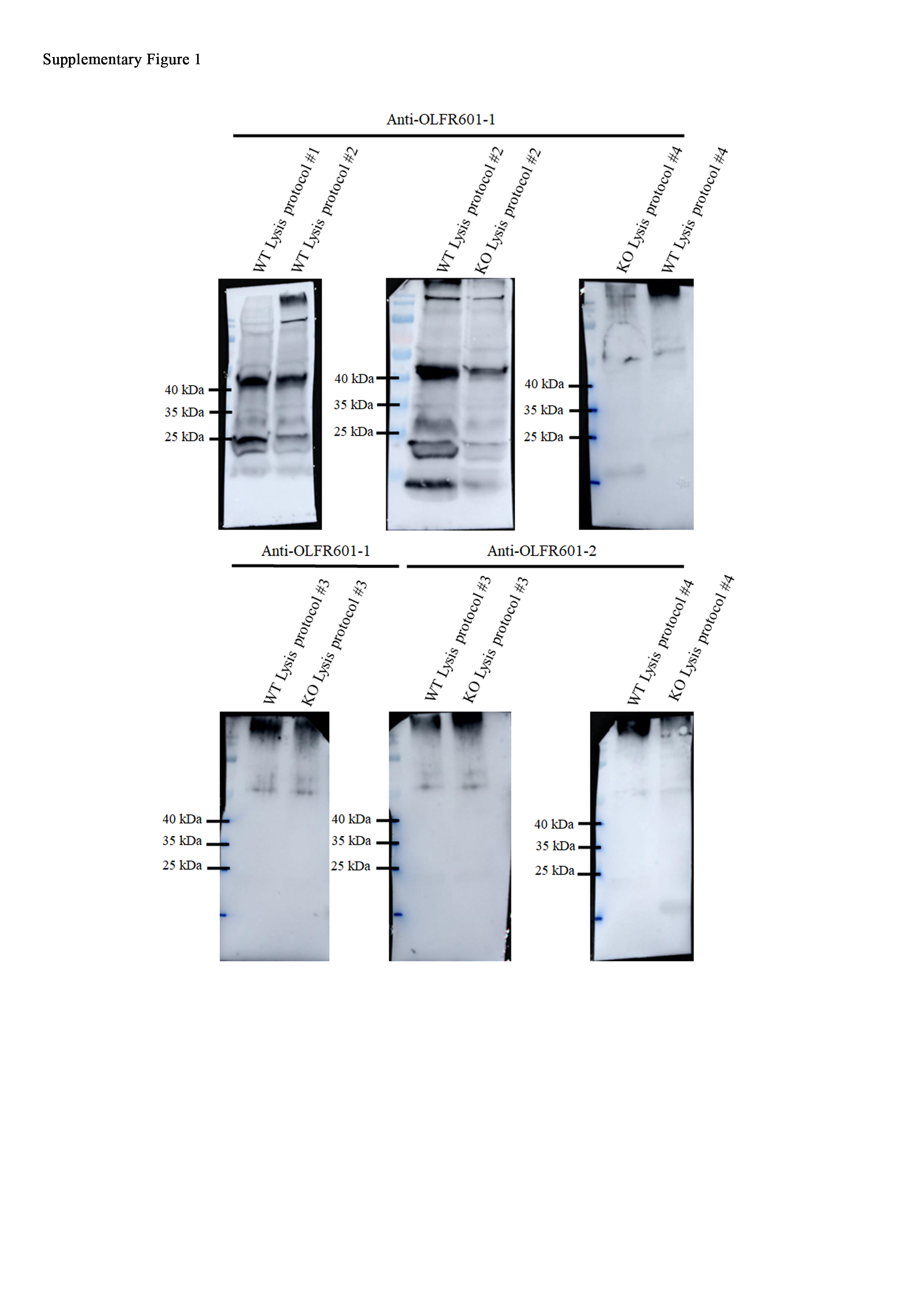

Supplement: Supplementary file 4 [file Image1.TIF]
