# Supplementary material for: The Sperm Olfactory Receptor OLFR601 is Dispensable for Mouse Fertilization
Source: Front Cell Dev Biol. 2022 Jun 3;10:854115. doi: 10.3389/fcell.2022.854115 (PMC9204177; doi:10.3389/fcell.2022.854115)
Supplement: Supplementary file 5 [file Table3.DOCX]

**Supplementary Table 3.** Protein identity of OLFR601 and several sperm proteins involved in sperm-egg interaction between mouse, human and bovine.

| Mouse protein | Human and bovine ortholog | % Identity mouse vs. human | % Identity mouse vs. bovine |
| --- | --- | --- | --- |
| OLFR601 | None* | 55.5 % (OR52M1) | 84.6 % (OR52S6) |
| IZUMO1 | IZUMO1 | 50.6 % | 48.6 % |
| SPACA6 | SPACA6 | 64.0 % | 62.2 % |
| TMEM95 | TMEM95 | 62.8 % | 61.7 % |
| FIMP | FIMP | 65.2 % | 52.5 % |
| SOF1 (LLCFC1) | SOF1 (LLCFC1) | 47.2 % | 59.6 % |
| DCST1 | DCST1 | 71.8 % | 73.0 % |
| DCST2 | DCST2 | 74.2 % | 73.6 % |

* For protein identity, the nearest paralog was considered.
